# The Connection between the Presence of Melanoma and Changes in Fibre Diffraction Patterns

**DOI:** 10.3390/cancers2021155

**Published:** 2010-06-04

**Authors:** Veronica J. James, Nigel Kirby

**Affiliations:** 1 Research School of Chemistry, Australian National University, Canberra ACT 0200, Australia; 2 Australian Synchrotron, 800 Blackburn Rd Clayton, VIC 3168, Australia; E-Mail: nigel.kirby@synchrotron.org.au

**Keywords:** melanoma, diagnosis, x-ray diffraction, skin

## Abstract

An accurate diagnosis of melanomas at an early stage correlates directly with a better prognosis. However the incidence of melanoma is still increasing along with the number of related deaths. Melanoma cells grow extremely fast, with the result that many patients present after metastasis has occurred, too late for effective treatment. This paper describes the changes in the fibre diffraction patterns of skin that indicate the presence of a melanoma. Identification of these changes would provide an alternative early low-cost, reliable diagnostic test which could be conducted on a regular basis in local radiology facilities using rotating anode X-ray generators or as a mass screening test using suitable small angle x-ray beam-lines at synchrotrons.

## 1. Introduction

Melanomas are the most potentially lethal of all the skin cancers. Although major changes have been made in the surgical procedures and in the therapy for melanoma, the incidence of this skin cancer is still increasing. These changes eliminate the need for grafts thereby greatly reducing hospital and recovery time. However they do not appear to improve the survival rate [1,2]. Diagnosis of all four types of melanoma at an early stage is the key to the best prognosis and to possibly preventing death. This requires that the tumour be diagnosed before it has reached Grade 4, infiltrated the lymph nodes or metastasised to other body organs. Since the most common type, the Superficial Spreading Melanomas, can grow extremely fast and the Nodular, Lentigo Maligna and Aeral Lentiginous melanomas are not easily recognised in the early stages, many patients present too late. In this paper we present a possible alternative diagnostic test using fibre x-ray diffraction of a skin biopsy. 

This use of x-rays is completely different from the familiar standard shadow patterns from routine medical examinations where the full wavelength spectrum of hard x-rays [energy range 20 to 120 keV (0.10 to 0.010 nm wavelength)] are shot through the body area of concern, hence the accepted metonymical use of the word x-ray to refer to a radiographic image so produced. Because bones absorb more energy than the soft tissue, the latent image on the film shows white areas for the bones, dark areas for the less dense tissue, but the pattern is clearly identifiable to the viewer.

In contrast, x-ray diffraction patterns bear no visual resemblance to the sample. Such patterns are produced by the scattering of a finely focussed monochromatic x-ray beam from the electron density distribution within the samples. Hard x-ray wavelengths are approximately the same length as the distances between atoms, (carbon-carbon and carbon-oxygen bond-lengths are ~0.15 nm), a requirement for the production of diffraction patterns, and are therefore ideal for diffraction studies of materials. The specific wavelength for the monochromatic beam produced by a rotating anode is determined by the material composition of the anode. Synchrotron x-radiation has many advantages such as vastly increased flux and brilliance, and the choice of a specific wavelength for an experiment, the latter is simply selected using a monochromator. Time for exposures on a synchrotron is greatly reduced from those required when using rotating anodes (days/hours to minutes/seconds) as a result of the increased flux.

Using such a beam the molecular structure of any crystalline material can be determined. The atoms in a crystalline material are arranged in regular lattices. For such crystals, the interference/diffraction patterns obtained are made up of a series of spots, as the crystal acts like a 3-D diffraction grating. The positions and the intensities of these spots can be measured and then using standard computational procedures the molecular structure can be determined. Since the atoms of any given crystalline substance are arranged in a pattern specific to that substance, any crystal of a particular substance will always give the same diffraction pattern, regardless of the location or X-ray source used. These comments apply equally to any crystals. The quality of the pattern will vary only with the quality of the beam and the selection and loading of the crystallised sample. Any change in the chemical composition of the substance, even the addition or subtraction of one atom, will cause a change in the pattern observed as the molecular structure is altered.

**Figure 1:**
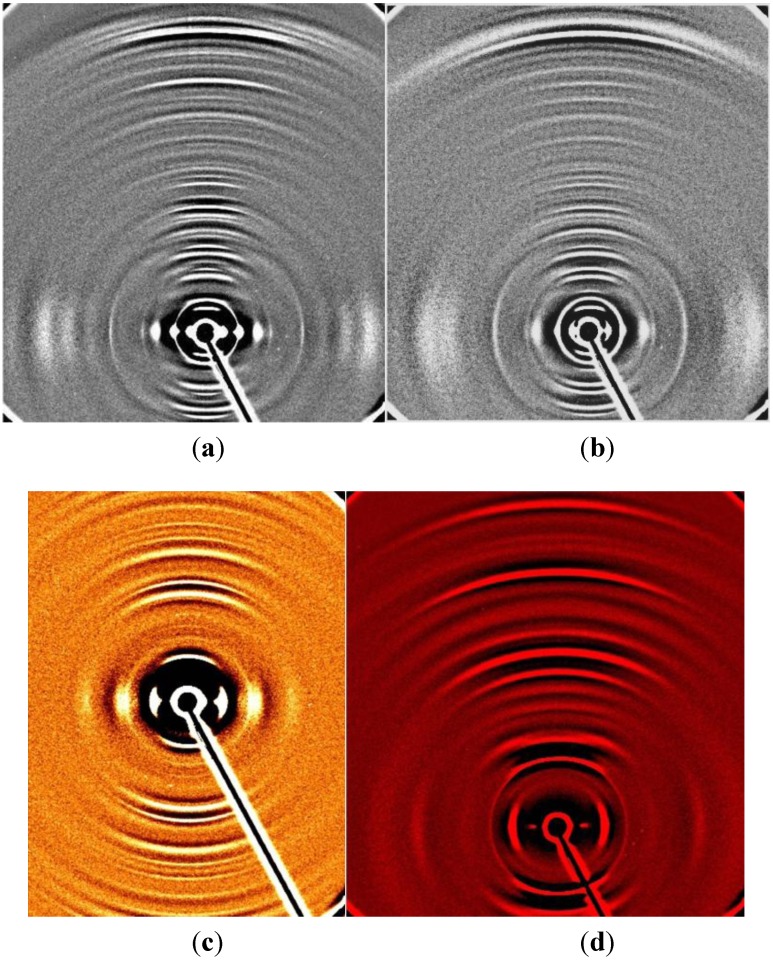
**(a)** This diffraction pattern was obtained from a cat’s whisker. Whiskers are naturally occurring pseudo crystalline, the vertical or meridional pattern results from the repeat distances along the whiskers. The dots and arcs in the equatorial pattern allow us to calculate the radii and centre to centre distances of the cylindrical components of the hard alpha keratin molecule. **(b)** This diffraction pattern was obtained from a pig’s hair. Although not as detailed as the whisker, it is still very crystalline as can be seen by the length of the meridional arcs. **(c)** All diffraction patterns are symmetrical across the centre as is seen in this pattern from an echidna quill. This is very useful in eliminating spurious peaks. **(d)** This pattern from a magpie’s feather illustrates the different molecular arrangement of the molecules in avian keratin from that of the hard α-keratin of whiskers, hair, quills and horns.

Similar comments apply to natural pseudo-crystalline materials. A comparison of the excellent pattern obtained by Fraser and McCrae [3] using a laboratory x-ray source with that obtained by Wilk *et al*. [4] using a synchrotron source illustrates that the quality is not altered by the source of x-rays, only the intensity of the pattern and the exposure times are altered. Changes in the diffraction pattern by alteration of the molecular arrangement or structure are also evident in the patterns of pseudo-crystalline materials such as the patterns obtained from keratinous materials, e.g., hair, quills, and feathers, Figure 1a, Figure 1b, Figure 1c and Figure 1d. Changes in these patterns have already been shown to occur if the hair is taken from any mammal with breast or colon cancer [5,6], or horses or humans with Alzheimer’s disease [7].

Many reports of the changes in x-ray diffraction patterns of human collagenous tissue which correlate with diseases of the particular tissues have been reported over the last 40 years. These include changes in tendon with insulin dependent diabetes [8], changes between normal and myxomatous heart valves [9], changes in bone with hereditary disorders such as brittle bone syndrome [10,11], changes in breast tissue with ageing and cancer [12], changes in skin with breast cancer, prostate cancer, [13,14], changes in corneal tissue with disease [15,16].

In order to obtain diffraction patterns of any tissue, the molecules must be arranged in an ordered parallel array, as for example the arrangement of collagen in tendon or keratin in hair. Good patterns from other tissues can be obtained by careful selection of the sample, such as the collagen aligned along the ducts in the breast. For better patterns of skin therefore, only the collagen plaques from the dermal layer are used, the epithelial and epidermal layers are scraped off as all such amorphous tissue contributes only to noise.

**Figure 2:**
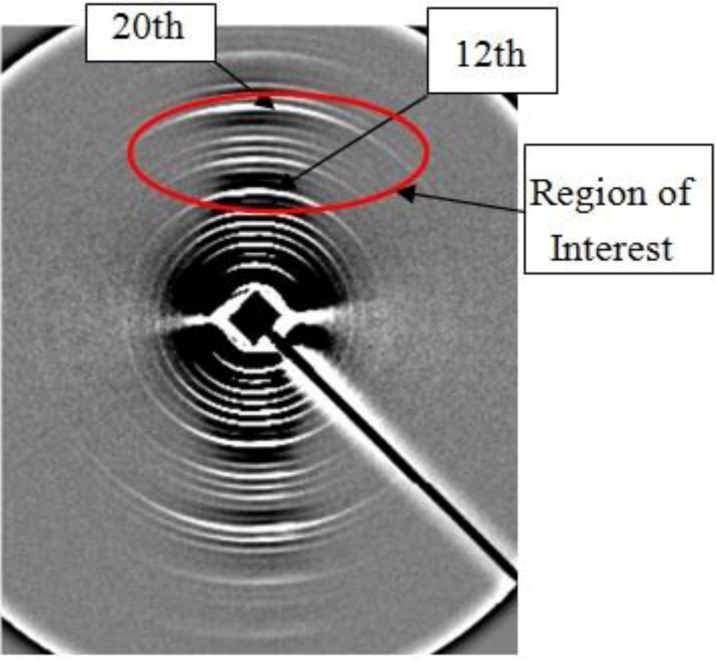
A diffraction pattern of the dermal collagen from normal skin. The meridional (vertical) reflections are smeared into arcs indicating that, although the 2D collagen fibres are somewhat aligned by gently stretching, they cannot be completely aligned. However the region of interest, for determining the possible presence of the change associated with melanoma, is clear of rings as the basic reflections are less intense and therefore are arcs and not complete rings.

The collagen plaques in the dermal layer of the skin are made up of a two dimensional crimped arrangement of heterogeneous helices containing collagen I, collagen III, and collagen V. When stretched these molecules preferentially align in the direction of the stretch. This alignment is never complete and therefore the meridional reflections are not as discrete as those of tendon but are smeared into arcs. The strong inner peaks become circles, shown in Figure 2. These do not impact on the analysis of the patterns observed in various cancers, as they fall inside the area of interest. However, if the whole diffraction pattern degenerates into rings, a repeat experiment with a new sample is required.

The specific changes that appear in skin for patients with breast cancer or prostate cancer [13,14] appear in the diffraction patterns of all skin samples, whether these samples are collected from skin adjacent to the site of the tumour or not. Samples of underarm skin were use in diagnosing the presence of breast cancer [13], and from the upper torso in prostate cancer. The changes observed are specific to the particular cancer but have been recorded at different intensities for different cancer grades e.g. grade 1 breast cancer gives a very weak ring by comparison with the more intense rings associated with more advanced carcinomas.

These changes only indicate the presence of the particular carcinoma. The actual location must then be determined by routine procedures. However, since the changes are specific to the different carcinomas, the type of carcinoma is clearly defined for each sample. No alternative external easily harvested tissue has been found for the diagnosis of melanoma [15]. Whilst changes in the x-ray pattern with melanoma have been reported [14], all samples for that study were taken from skin adjacent to the melanoma. This paper reports that this change extends into the area around the melanoma which is regarded as normal that is well outside the now recommended excision margins. Some of these patients presented for new cancers at the same site three years later.

## 2. Results and Discussion

**Figure 3:**
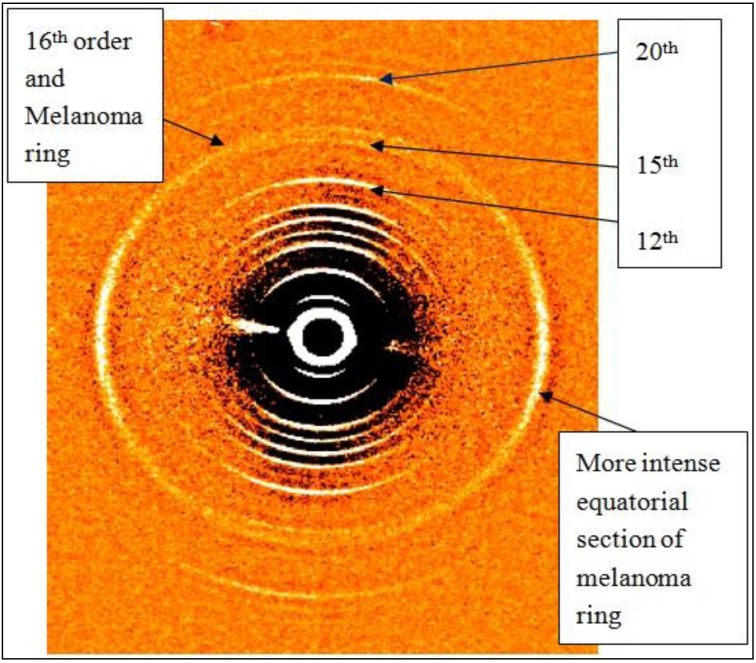
The diffraction pattern shown here is the pattern obtained from persons with melanoma. The normally stronger 12th, 15th and 20th meridional reflections of skin collagen are indicated and in addition the normally weaker 16th order which coincides with the melanoma ring is shown. This additional ring is, as usual, far more intense in the equatorial direction.

A full description of the normal pattern for skin collagen [18,19] and the clear and consistent change in this pattern in the presence of melanoma has been given elsewhere [14], the first two of these publications being based on rotating anode data, the third on synchrotron data. For the purpose of using this diffraction technique to diagnose melanoma, only the change in or additions to the normal diffraction pattern have to be evaluated. The specific change in the normal skin pattern for persons with melanoma is an additional ring which superimposes on the 16th meridional collagen reflection, (spacing of 4.08 ± 0.03 nm). This ring is not uniform in intensity but is more intense in the equatorial direction as is evident in Figure 3. For some patients, only the equatorial arcs are present.

To determine the margin of this change in relation to the site of the tumour, a set of blinded samples supplied for our study included two samples of skin from each patient. The first was taken from the area adjacent to the tumour. The second was taken from an area remote from the tumour, outside the accepted excision margin. The results of this study are given in Table 1. These results indicate that the change exists beyond the normally accepted excision margin.

**Table 1:** Diffraction results of a set of samples that included sections close to and remote from the melanoma site indicating that those patients who had recurrence of tumours at the same site had similar patterns in both samples.

No	Date of birth	Site	Date collected	Adjacent to carcinoma	Distant from carcinoma	Recurrence same area
1	1.11.1979	right auxiliary	19.5.2006	equatorial arcs	very weak ring	
2	15.10.1956	right thigh	23.6.2006	very weak ring	equatorial arcs/rings	
3	20.7.1940	right groin	16.6.2006	weak diffuse ring	very weak arcs	yes
4	26.1.1953	multiple	22.8.2006	ring	weak ring	yes
5	23.8.1966	chest/back torso	26.7.2006	Ring + unusual pattern	Ring + unusual pattern	yes
6	16.6.1923	right post auxiliary	14.11.2006	diffuse ring	ring	yes
7	13.6.1925	right upper arm	3.11.2006	fuzzy ring	normal	
8	15.4.1945	right buttock	12.2.2009	weak ring	ring	
9	12.9.1950	right leg	17.2.2009	ring	very weak ring	
10	23.4.1959	left thigh	26.3.2009	no sample	strong arcs equatorial	
11	11.8.1934	right buttock	27.2.2009	no sample	weak ring	yes

**Figure 4:**
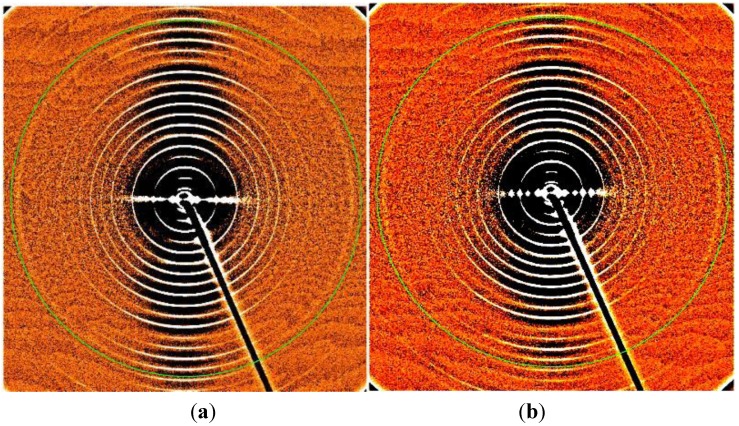
**(a) & (b)** Figure 4(a) and Figure 4(b) below are two of the patterns from along the radial section of a segment taken from a surgical section. This segment was cut so that the point of the arc was near the melanoma in situ and the arc was at a distance of 4cm from the tumour. These pictures and all those between these extremes clearly confirm that the molecular change in the skin is spread across the entire section. The green rings on 17^th^ order have been added to assist in the location of the weak melanoma passing just inside it through the 16^th^ order.

To verify this result, a sector was cut from the circular section of tissue (radius 4 cm) removed with a melanoma, with the point nearest the tumour. Studies were made at 2 mm intervals along three radii of this segment. Figure 4 shows two of these patterns. Figure 4(a) is the diffraction pattern of skin taken from the area adjacent to the melanoma. Figure 4(b) is from the pattern from skin taken from the periphery of the arc. No difference was found in the 60 patterns obtained from points across this segment.

Although these results are based on very few samples, the findings here are consistent with the results obtained with other cancers where changes in skin have been consistently identified in areas remote from the cancer site [13]. If this is not so, it may be important to remove all changed tissue as in the case of changes in the colon cancer where there is a high probability that a later cancer will occur at that site [6]. Our results reported here indicate that there has been a recurrence of a further melanoma at these sites but further studies are underway to evaluate this possibility.

**Figure 5:**
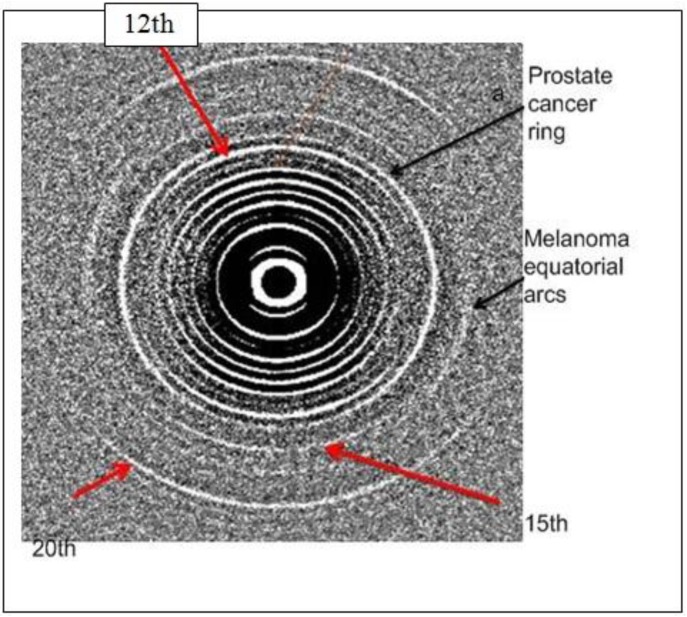
This diffraction pattern of skin taken from a surgical section for a melanoma removal from above the waist on the back shows clearly the presence of both melanoma and prostate cancer. The 12th, 15th and 20th orders are indicated along with the prostate cancer ring and the melanoma arcs which are far more intense in the equatorial direction.

Our studies have also confirmed that if the patient has more than one type of cancer, the specific changes associated with each type will be present in the final diffraction pattern. An example of this is given in Figure 5, where both the changes for prostate cancer and melanoma are identified.

**Figure 6:**
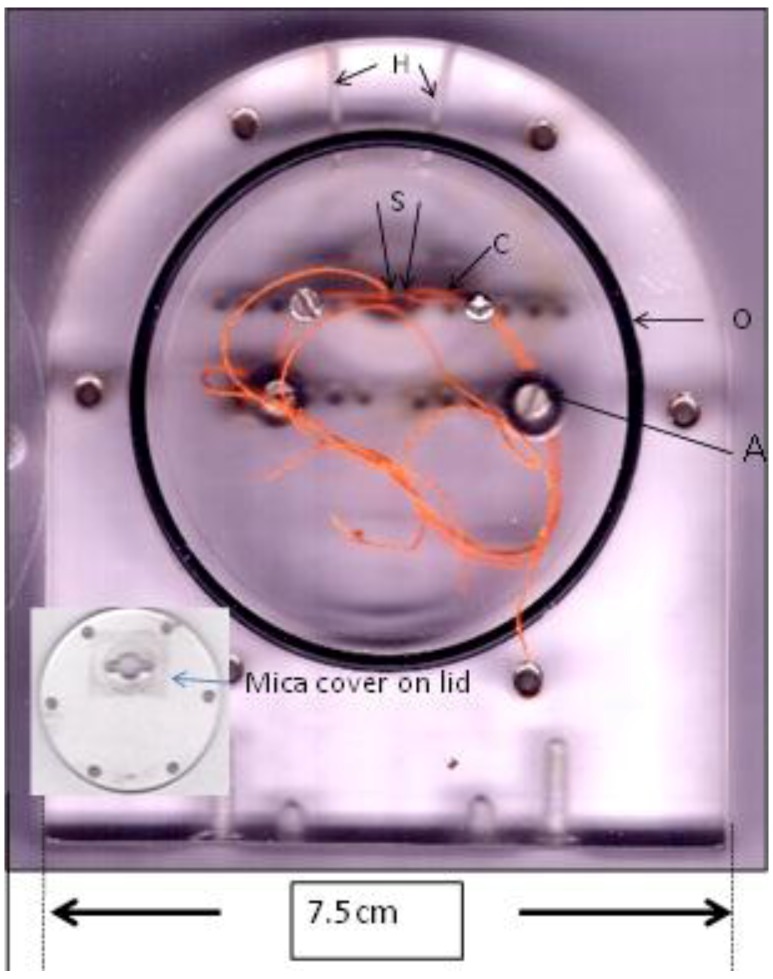
A loaded sample cell for obtaining a diffraction pattern from a skin sample, S, measuring (0.5 mm × 3 mm) after stretching by carefully winding and clamping the screws [A] to which the sutures (c) are attached. The two 1mm holes allow water to be inserted into the cell after loading and sealing. The lid is sealed with the O-ring, (O), and lid (insert). The x-rays pass through the Mylar window which covers the sample section of the cell

## 3. Experimental Section

A total of 52 skin samples taken from melanoma patients were included in the 296 blinded skin samples that have been examined in this ongoing research project. The age range for these samples was between 18 and 90. These have included 211 controls. Some of these skin samples were obtained from routine surgery or from necropsies and these were typically small sections, 1mmx5mm approximately. Alternatively, 3 mm full punch biopsies of skin were collected with the consent of the patient. Ethics approval for all experiments and procedures has been granted at all collection points. 

Immediately after excision, the samples were placed in physiological saline and stored at −20 °C until required. Before mounting in the cells, which have been specifically designed to maintain 100% humidity throughout the experiment, the skin samples were gently scraped to remove the epithelial and epidermal layers to expose the dermal layer, at all time maintaining 100% humidity by keeping the sample submerged in the buffer. Small sections are removed from the collagen plaques and sutures are attached to both ends to assist with mounting to the experimental cells, Fig 6. These cells have the capability to stretch samples slightly to remove the natural crimp in the collagen fibres. The collagen is aligned preferentially in the direction of the applied stretch thus giving rise to the meridional (vertical) pattern, Figure 3. The meridional lattice spacing for wet skin from controls is 65.2 nm [18,19]. The equatorial (horizontal) pattern reflects the cylindrical packing arrangement.

The skin samples produced excellent diffraction patterns. All samples yielded patterns which varied only in the equatorial direction over the age range of samples used in this study. Diffraction experiments were conducting using the Rotating Anode X-ray generator at the Rex Vowels Low Angle Diffraction Laboratory, UNSW, and at Rigaku Corporation Japan with sample to detector distances of 200 mm and 1000 mm. Synchrotron studies were carried out on the BL15A Photon Factory, Tsukuba, Japan, at the BioCAT Facility, the Advanced Photon Source (APS), Argonne, USA and at the SAXS-WAXS Beam line at the Australian Synchrotron, Melbourne. Sample to detector distances at the synchrotrons varied between 400 mm and 1300 mm at the various facilities. Calibration was achieved by comparison with diffraction patterns from moist rat-tail tendon or by comparison with a silver behenate sample (first order spacing: 5.838 nm) at the synchrotrons. The synchrotron fibre diffraction intensity distributions were recorded on FUJI BASIII imaging plates at the Photon Factory and at BioCAT and the data was extracted by electronic scan. A MAR165 detector was used on-line on BioCAT and at the Australian Synchrotron to aid in the alignment of the sample and also for data collection. Initial analyses were carried out using a combination of MATLAB-R-2007 and ProcessFITS or SAX15ID. This was followed by a high precision analysis of the two dimensional data using the astronomy packages IRAF (1986) and SAO Image (1991). These Smithsonian packages have been designed for viewing stars over a wide range of intensities from the very weak to the very strong by continually limiting the intensity range under observation. They are, therefore, perfectly designed for handling the wide dynamic range of intensities obtained from the imaging plates and the MAR165. In addition, intensity/position plots (implots) can be displayed and printed out along any designated line. This is especially important as changes associated with Grade 1 tumours can be extremely weak and not apparent using other computer programs. Depending on the quality of the data, different methods available in both IRAF and MATLAB were used in the analysis. These included curve fitting which was extended to second order derivative plots to locate ill defined or weak peaks [20]. The background removal was achieved using one of the IRAF packages, BOXCAR, a standard mathematical boxcar data smoothing procedure. Different box-sizes were used depending on the overall distribution of the particular patterns or over sections of a pattern. A detailed description of this process is given in [4].

## 4. Conclusions

Our results to date indicate that diffraction studies of skin could provide a diagnostic test for melanoma. Only one test is required whether one or more types of cancer are present as the specific change for each cancer present will be identifiable on the x-ray. As an added advantage all such skin diagnostic tests could be routinely studied at a reasonable time-rate and personnel cost in local radiology units, thus removing the need for transportation to remote facilities. The specimens can be harvested at local facilities, since samples do not deteriorate with correct storage. Tests are continuing but at this stage insufficient samples have been studied to obtain statistically reliable specificities and sensitivities but our results would indicate that such tests would provide early, low-cost, yet reliable tests using this easily accessible biological tissue.
